# Enhancing the ENSO Predictability beyond the Spring Barrier

**DOI:** 10.1038/s41598-020-57853-7

**Published:** 2020-01-22

**Authors:** Han-Ching Chen, Yu-Heng Tseng, Zeng-Zhen Hu, Ruiqiang Ding

**Affiliations:** 1 Department of Atmospheric Sciences, University of Hawaii at Manoa, Honolulu, USA; 2 Institute of Oceanography, National Taiwan University, Taipei, Taiwan; 3 NOAA/NWS/NCEP/Climate Prediction Center, College Park, Maryland, USA; 4 State Key Laboratory of Earth Surface Processes and Resource Ecology, Beijing Normal University, Beijing, China

**Keywords:** Atmospheric science, Climate change, Ocean sciences

## Abstract

El Niño-Southern Oscillation (ENSO) is the dominant interseasonal–interannual variability in the tropical Pacific and substantial efforts have been dedicated to predicting its occurrence and variability because of its extensive global impacts. However, ENSO predictability has been reduced in the 21^st^ century, and the impact of extratropical atmosphere on the tropics has intensified during the past 2 decades, making the ENSO more complicated and harder to predict. Here, by combining tropical preconditions/ocean–atmosphere interaction with extratropical precursors, we provide a novel approach to noticeably increase the ENSO prediction skill beyond the spring predictability barrier. The success of increasing the prediction skill results mainly from the longer lead-time of the extratropical–tropical ocean-to-atmosphere interaction process, especially for the first 2 decades of the 21^st^ century.

## Introduction

The El Niño-Southern Oscillation (ENSO) is the dominant interseasonal–interannual variability in the tropical Pacific and it exerts significant influence on weather and climate all over the world via atmospheric teleconnection^1^. Because of its global influence on the atmosphere and oceans, ENSO directly affects worldwide human life and the terrestrial and marine ecosystems. Substantial efforts have been dedicated to developing different forecast approaches to predict ENSO evolution several seasons in advance^2–5^. Previous studies used the Warm Water Volume (WWV) along the equatorial Pacific as a key precursor of ENSO^6–9^. However, WWV anomalies are not the only requirement for ENSO development^1,10^. It has been suggested that in addition to WWV condition, a series of westerly wind events/easterly wind surges (WWEs/EWSs) in the western–central Pacific play an important role in the onset and maintaining of ENSO events^11–14^.

Interestingly, the ENSO forecast skill was noticeably reduced after 2000 in terms of the correlation and root mean square error (RMSE) (e.g., failures of forecast in 2012/13 and 2014/15 are good examples; most models predicted a strong ENSO event but these years ended up with a neutral or a weak El Niño conditions). Consistently, the strength of the seasonal footprinting mechanism has intensified during the past 2 decades^15^. The footprinting mechanism can trigger central Pacific types of ENSO events via the subtropical ocean–atmosphere coupling^16–20^. The amplified footprinting mechanism makes the ENSO more complicated and harder to predict because of changes in ENSO frequency and magnitude in the 21^st^ century^1,15^. On interseasonal time scales, the spring predictability barrier (SPB) is still a large challenge that limits ENSO predictability, which tends to cause the model’s forecasting skill to decrease sharply when the prediction is made through spring^21,22^.

**Figure 1:**
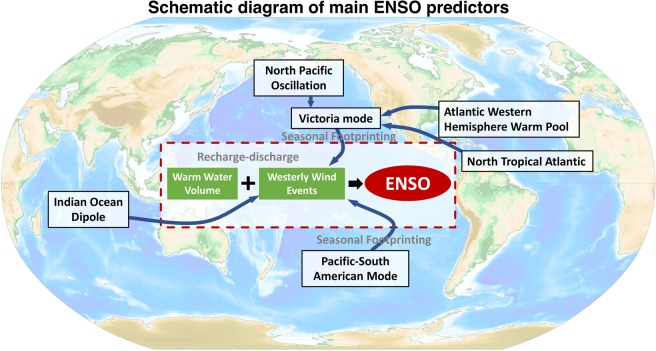
Schematic diagram of main ENSO predictors. The topographical mapping is based on 2‐Minute Gridded Global Relief Data (ETOPO2) topography and generated using Matlab R2018b. The Matlab script (https://www.asu.cas.cz/~bezdek/vyzkum/rotating_3d_globe/index.php#1b) is provided from Bezdek and Sebera (2013)^69^.

Recently, some statistical models based on the concurrent tropical oceanic and atmospheric conditions have been used to predict ENSO evolution with skillful prediction^23–25^. These models outperformed some dynamic models and suggested key roles for subsurface heat content evolution together with the modulation of WWEs/EWSs over the central Pacific to improve ENSO predictability compared to that solely based on the WWV. Although the equatorial atmosphere and ocean coupling is crucial in ENSO evolution, the prediction skill of ENSO is limited if the predictors are only confined to the tropical or subtropical Pacific Ocean^1,26^. ENSO evolution does not only rely on the tropical Pacific^23,27–29^. Some studies have suggested that the extratropical Pacific sea level pressure (SLP) anomalies (SLPAs) during precedent winter over both the North and South Pacific, independent of the low-frequency WWV contribution, can modulate ENSO evolution via a footprinting-like mechanism^1,15–18,30^. In addition, precursory signals from other oceans, such as the Atlantic and Indian Oceans, can also help to trigger ENSO events via atmospheric teleconnection^31,32^. Possible ENSO key predictors and their influencing routes are summarized in Fig. 1.

To enhance the reliability in predicting ENSO events, here we will include both tropical (WWV, WWEs/EWSs) and extratropical factors and their interactions. Thus, a novel ENSO prediction model (*EPM*) combining the tropical status and extratropical ocean–atmosphere interaction is derived here to demonstrate the success in increasing 6- to 10-month lead prediction skill beyond the SPB and to explain the dynamics behind the model. This improvement was especially noticeable in the first 2 decades of the 21^st^ century.

## Results

### Impact of extratropical atmospheric forcing on ENSO

Previous studies suggested that the extratropical atmospheric forcing is mostly a stochastic process^16,17,33^. However, some recent observational and modeling studies have suggested that the atmospheric variability outside the tropical Pacific has a significant influence on the onset of ENSO events, including the impact of atmospheric variability over the North Pacific^16–20,27,34–36^ and the South Pacific^37–41^. Signals in these extratropical atmospheric variabilities have been found to be useful as a major precursor for ENSO occurrence^16,28,29,36,38,41,42^.

**Figure 2:**
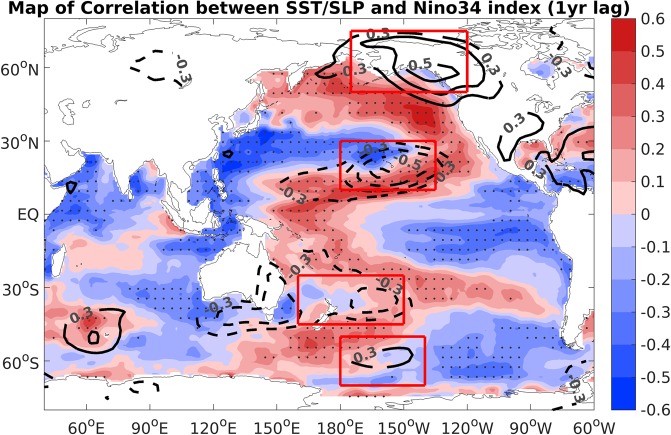
Correlation map of boreal winter (November–March) SST (shading) and SLP (contours) anomalies with the Niño3.4 index during the following boreal winter. The black dots indicate the correlation of SSTAs exceeding a 90% confidence level using a t-test. The SLPAs with correlations significant above the 90% confidence level are also shown. The four red boxes (from top to bottom: 175°–120°W, 50°–75°N and 180°–135°W, 10°–30°N and 160°E–150°W, 25°–45°S and 180°–140°W, 50°–70°S) are used to defined the extratropical forcing index (*EP*_*EX*_).

Figure 2 shows the correlations of winter (November–March) sea surface temperature (SST) and SLP anomalies with the Niño3.4 index (defined as the averaged SST anomalies (SSTAs) over the region 170°–120°W, 5°S–5°N) 1 year later. A significant SLPA precursor signal appeared in both the North and South Pacific. The SLPA precursor signal and corresponding SSTAs in the North Pacific presented a typical North Pacific Oscillation (NPO)-like pattern^43,44^, a north–south dipole of SLPAs, whereas the SLPAs and SSTAs in the South Pacific show a pattern similar to the Pacific–South American pattern (PSA)^45^, characterized by a wave-like pattern of SLPAs. To identify the major impact of the extratropical ENSO precursors, the impacts of these two dipole modes over the North and South Pacific can be combined to construct an ENSO predictor according to extratropical teleconnection (*EP*_*EX*_) (see Methods).

**Figure 3:**
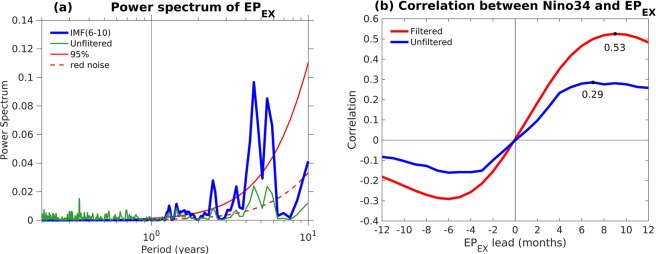
(**a**) Power spectrum analysis of the unfiltered (green curve) and EEMD filtered (IMF_6–10_; blue curve) *EP*_*EX*_. Red noise (dashed red curve) and the 95% statistical confidence (solid red lines) for the IMF_6–10_ of *EP*_*EX*_ are indicated. The vertical black line represents a period of 12 months. (**b**) Lead-lag correlation of Niño3.4 index with the unfiltered (blue curve) and EEMD filtered (red curve) *EP*_*EX*_. Maximum correlations are labeled (black dots).

Figure 3a illustrates the power spectrum of unfiltered *EP*_*EX*_ index anomalies that were calculated via Fast Fourier Transform (green curve). We note that the *EP*_*EX*_ index can be separated into two distinct time scales. The longer time scale is dominated by interannual variability with periods varying from 12 months to 7 years^46,47^. The other time scale exhibits higher frequency with a period of less than 12 months and it was associated with mid- and high-latitude stochastic variability^16,17,33^. To remove the high-frequency component and maintain the interannual extratropical variability, an Ensemble Empirical Mode Decomposition (EEMD) (see Methods) is applied to decompose the *EP*_*EX*_ index with different frequencies. The IMF_1-5_ of *EP*_*EX*_ is composed of high frequencies (not shown) and the IMF_6–10_ of *EP*_*EX*_ exhibits a lower frequency with a period from 12 months to 7 years (blue curve in Fig. 3a). The lead-lag correlation of the monthly EEMD filtered and unfiltered *EP*_*EX*_ index, respectively, with the monthly Niño34 index is shown in Fig. 3b. For lead times of 6–10 months, the EEMD filtered (unfiltered) *EP*_*EX*_ index shows a significant correlation with the Niño34 index, with a peak at a lead time of approximately 9 (7) months^29^. The maximum correlation coefficient between the Niño34 index and the EEMD filtered *EP*_*EX*_ (0.53) is clearly larger than that of the unfiltered *EP*_*EX*_ (0.29). The relationship between ENSO and extratropical forcing is significant on the annual-to-interannual time scales, suggesting a potential of the extratropics to enhance the predictability of ENSO. Moreover, the impact of extratropical forcing varies strongly with seasons. The most significant impact of extratropical forcing on the ENSO is in MAM (Fig. S1), consistent with the time scale discussed above.

**Figure 4:**
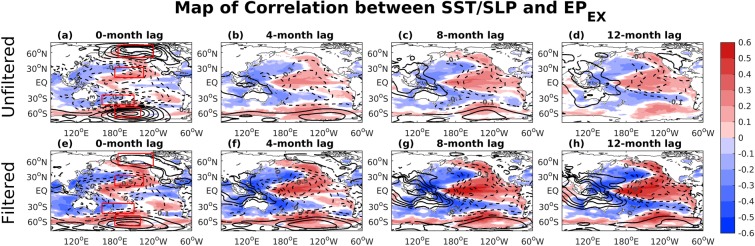
Correlations of the SST (shading) and SLP (contours) anomalies based on the (**a–d**) unfiltered and (**e–h**) EEMD filtered *EP*_*EX*_ at different lag time (SST and SLP anomalies lag *EP*_*EX*_). Only the correlations significant at or above the 95% confidence level are shown.

To further demonstrate the impact of extratropical atmospheric variability on the ENSO variability, the lag correlations of SSTAs and SLPAs with the monthly EEMD filtered and unfiltered *EP*_*EX*_ index are shown in Fig. 4. The lag correlation patterns of SSTAs and SLPAs are both similar to the SSTAs and SLPAs that are associated with ENSO^28^, and the relationship is stronger for the EEMD filtered *EP*_*EX*_ than for the unfiltered *EP*_*EX*_. These results support the previous arguments that the NPO-like or PSA-like atmospheric pattern associated with surface wind fields over the North or South Pacific can modulate SSTAs in the tropical Pacific and alter the ENSO evolution, as shown in the evolution of Fig. 4a–d (also e–h)^16,17,27,29,36,41^.

The subtropical SST pattern in the Northern Hemisphere is the typical footprinting of the Victoria mode (also called the Pacific meridional mode, PMM, for a regional domain)^48–50^ while the quadrupole SST pattern in the Southern Hemisphere is called the South Pacific meridional mode^40,41,51^. These SST patterns may persist until boreal summer (hereafter, the seasons referred to are those of the Northern Hemisphere) and subsequently increase the zonal SST gradient across the western–central tropical Pacific and then strengthen the anomalous westerlies (easterlies) along the equatorial Pacific to initiate an El Niño (La Niña) event^29,41^ (see the evolution in Fig. 4b–c,f–g, respectively). Finally, the equatorial Pacific SSTAs reach the peak as an ENSO event with a corresponding west–east dipole of SLPAs (called Southern Oscillation) after about 8 months, consistent with the lead-lag relationship in Fig. 3b (*EP*_*EX*_ leads Niño34 index by 6–10 months)^29^. These results support that the extratropical teleconnection can be an important precursor to trigger ENSO events and to potentially improve the ENSO predictability, specifically beyond the time scale of SPB.

### Predicting the ENSO beyond the Spring Predictability Barrier (SPB)

The WWV and WWEs/EWSs associated with key tropical Pacific dynamics have been considered to be essential processes for the ENSO occurrence^6,23,24^. However, the extratropical ocean–atmosphere interaction commonly plays an active role in the onset of ENSO events. To demonstrate these key processes in enhancing the prediction skill, we constructed a multivariate linear regression model based on the tropical dynamics and extratropical ocean–atmosphere interaction, as discussed in the Methods. For example, a 6-month lead multivariate linear regression model can be constructed as follows:

1 $$\begin{array}{c}EPM(t+6month)=\frac{{\sigma }_{O}}{{\sigma }_{P}}(0.43\,E{P}_{WWV}(t)+0.22\,E{P}_{OA}(t)+0.18\,E{P}_{EX}(t))\end{array}$$

**Figure 5:**
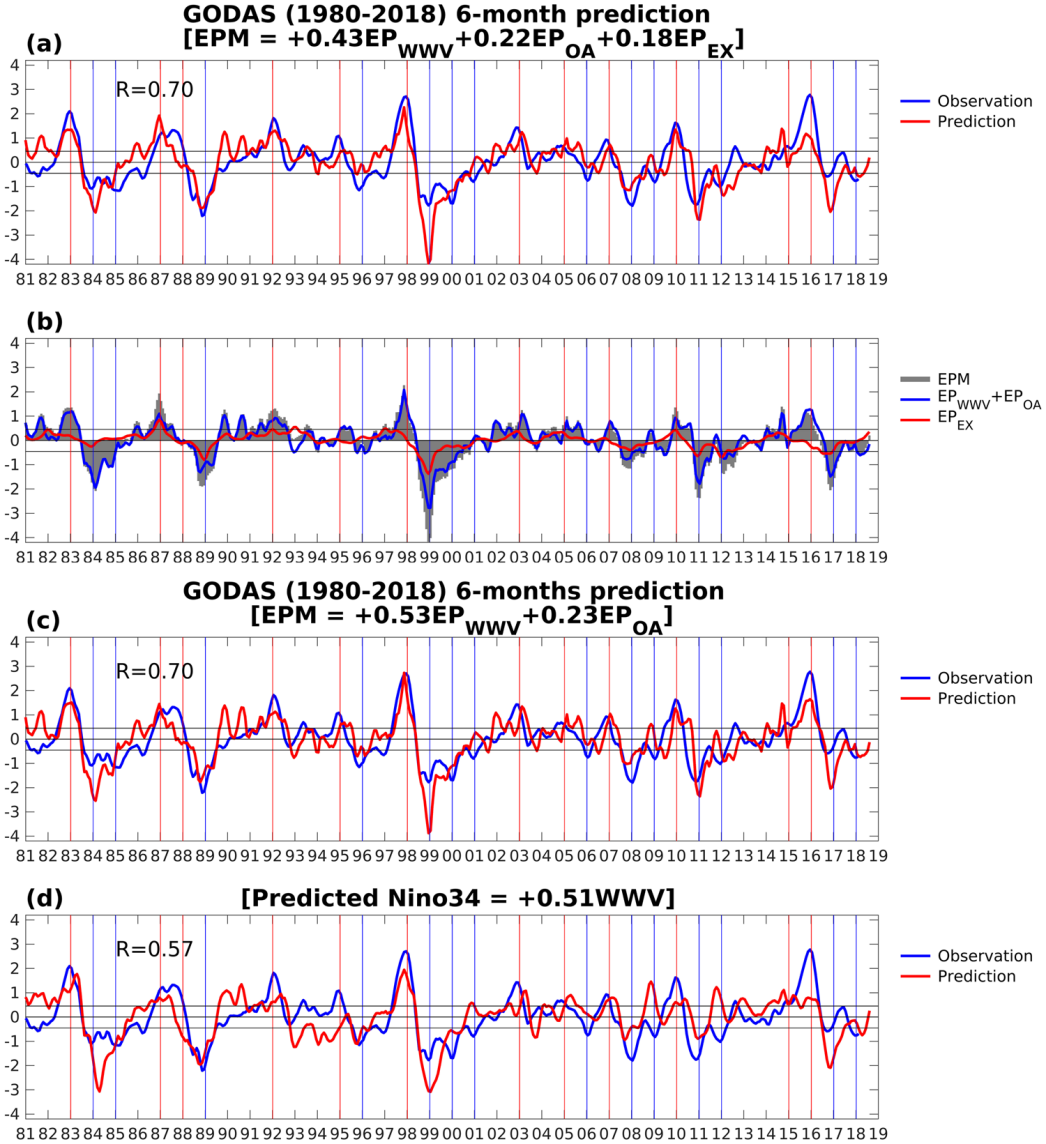
The 3-month running mean time series of the predicted Niño3.4 index (red line) generated by the 6-month lead regression model based on (**a**) the tropical and extratropical precursors together (**c**) the tropical dynamics (*EP*_*WWV*_ and *EP*_*OA*_ only) and (**d**) WWV index overlaid with the observed Niño3.4 index (blue line). The vertical lines represent the peak year of El Niño events (red lines) and La Niña events (blue lines). (**b**) The time series of predicted Niño3.4 index contributed by *EPM* (grey shading), *EP*_*WWV*_ + *EP*_*OA*_ (blue curve) and *EP*_*EX*_ (red curve).

Figure 5a shows a 3-month running mean time series of the monthly *EPM* (red line) using the 6-month lead linear regression model overlaid with the observed 3-month running mean Niño34 index (blue line). The correlation between predicted and observed Niño34 index was significant (R = 0.70) from 1981 to 2018. This 6-month lead hindcast skill was quantitatively similar to that based on the tropical condition only (Fig. 5c) and superior to that based on the WWV index (R = 0.57 in Fig. 5d). The WWV index was defined as the volume of water above 20 °C isotherm in the equatorial Pacific region (5°S–5°N, 120°E–80°W).

For the 6-month lead prediction, the relative contributions of the tropical dynamics and the extratropical influence on the *EPM* suggested the tropical dynamics associated with WWV and WWEs/EWSs (*EP*_*WWV*_ + *EP*_*OA*_) can capture most of the ENSO events (blue curve in Fig. 5b). The extratropical atmospheric influence (*EP*_*EX*_), in addition, can further provide useful information to enhance the hindcast skill (red curve in Fig. 5b). However, the improvement was limited, as expected. The *EP*_*EX*_ leads *EP*_*WWV*_ + *EP*_*OA*_ by about 2–3 months. The longer lead-time of the extratropical precursors suggests that the hindcast skill improvement is beyond 6-months, and thus potentially reduces the constraint of SPB.

To further examine the hindcast skill of *EPM*, we calculated the correlation and the RMSEs with different lead times in Table 1. Here, the *EPM* based on tropical dynamics only is presented as *EPM*_*TD*_, and the *EPM* considering tropical and extratropical precursors together is presented as *EPM*_*TD+EX*_. For all lead-time hindcast (6-month and longer), the *EPM*_*TD+EX*_ had higher hindcast skill in terms of correlation and RMSE measurements than *EPM*_*TD*_ and WWV index. The correlation of *EPM*_*TD+EX*_ was 0.65 (0.61) for an 8-month (10-month) lead, while the corresponding correlation was only 0.61 (0.52) for *EPM*_*TD*_ and 0.51 (0.44) for WWV index. The RMSE skill also suggested that the hindcast Niño34 index based on *EPM*_*TD+EX*_ was generally better than that based on either *EPM*_*TD*_ or the WWV index.

**Table 1:** The hindcast skills according to percentage correct, correlation, and RMSE between 3-month running mean observed and predicted Niño34 index based on the WWV index only, tropical dynamics only (*EPM*_*TD*_), tropical and extratropical precursors together (*EPM*_*TD+EX*_) with different lead-times for the period of 1980–2018.

Lead time	Percent correct (%) El Niño (La Niña)			False alarm rate (%) El Niño (La Niña)			Correlation			RMSE (^o^C)		
	*EPM*_*TD+EX*_	*EPM*_*TD*_	WWV	*EPM*_*TD+EX*_	*EPM*_*TD*_	WWV	*EPM*_*TD+EX*_	*EPM*_*TD*_	WWV	*EPM*_*TD+EX*_	*EPM*_*TD*_	WWV
Six-month	92 (86)	92 (79)	83 (71)	39 (14)	39 (21)	47 (23)	0.70	0.70	0.57	0.70	0.70	0.84
Eight-month	83 (79)	83 (64)	75 (64)	38 (15)	50 (36)	50 (36)	0.65	0.61	0.51	0.76	0.80	0.90
Ten-month	83 (71)	75 (71)	75 (64)	38 (17)	50 (41)	47 (31)	0.61	0.52	0.44	0.80	0.89	0.96

The percent correct is defined as the fraction of the observed events which are correctly predicted. The false alarm is defined as the fraction of the predicted events actually do not occur. The perfect score is 100% and 0% for percent correct and false alarm rate, respectively.

We also computed the percent correct metric, which is defined as the fraction of the observed events that are correctly predicted, in Table 1 to quantify the robustness of *EPM* for predicting ENSO events. Since 1980, the *EPM*_*TD+EX*_ with 6-month lead was 92% percent correct for El Niño and 86% percent correct for La Niña. The missed 1987/1988 El Niño, which was a double-dip El Niño after the 1986/87 El Niño event, was reinforced by the strong and uninterrupted WWEs with decaying heat content in the central tropical Pacific. For the missed 1995/96 La Niña, no significant EWSs and enhanced heat content exist with an unfavorable extratropical contribution during the developing phase. The missed 2005/06 La Niña was a weak event that was associated with poorly pre-conditioned WWV and weak EWSs.

The false alarm rate metric, defined as the fraction of the predicted events that do not actually occur, is also shown in Table 1. Generally, the performance of *EPM*_*TD+EX*_ was better than others in percent correct and false alarm metrics. The improvement in the hindcast skill of *EPM*_*TD+EX*_ was more significant for false alarms, but it only slightly increased the skill of the percent correct for *EPM*_*TD+EX*_. This is because the evolution of accumulated WWV is necessary for an ENSO event, and the prediction models based on equatorial heat content (*EPM*_*TD+EX*_, *EPM*_*TD*_, and WWV index) easily capture the occurrence of an ENSO event using the existing WWV signal. However, the evolution of an ENSO event does not solely rely on the WWV condition, and other factors, e.g. extratropical forcing, are also important to determine the occurrence of an ENSO event. That is why the hindcast skill of the false alarm rate can be improved significantly when the tropical and extratropical precursors are all considered in *EPM* (*EPM*_*TD+EX*_), compared with that only the tropical dynamics are included in *EPM* (*EPM*_*TD*_).

### *EPM* and hindcast skill in different prediction lead time

**Figure 6:**
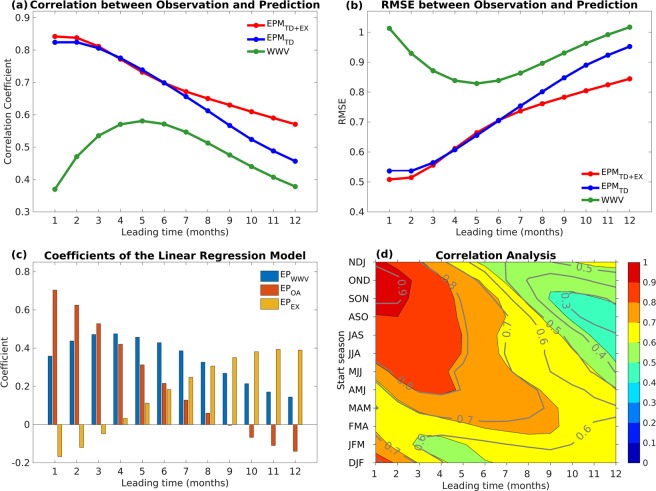
(**a**) Correlation and (**b**) RMSE between 3-month running mean observed and predicted Niño34 index based on the WWV index only (green curve), tropical dynamics only (*EPM*_*TD*_; blue curve), tropical and extratropical precursors together (*EPM*_*TD*+*EX*_; red curve) with different lead-times. (**c**) Coefficients of the multivariate linear regression model contributed by *EP*_*WWV*_ (blue bars), *EP*_*OA*_ (red bars) and *EP*_*EX*_ (yellow bars) with different leading times. (**d**) Correlation between 3-month running mean observed and predicted Niño3.4 index based on *EPM*_*TD+EX*_ (shadings), *EPM*_*TD*_ (contours) as a function of start month and lead time.

Hindcast skill is lower with a longer prediction lead time^4^. To quantify the influence of the tropical and extratropical precursors on the lead-time, we further analyzed the correlation and RMSE between the observed 3-month running mean and the predicted Niño34 index based on the WWV index only, tropical dynamics only (*EPM*_*TD*_), or tropical and extratropical precursors together (*EPM*_*TD*+*EX*_) with different lead-times (Fig. 6a,b). When we only consider the WWV index, the best hindcast skill appeared in the 5-month lead, which is consistent with the role of equatorial heat content in the recharge–discharge process^9,52^. If both of the WWV propagation and the ocean–atmosphere coupled feedback are included (*EPM*_*TD*_), the hindcast skill significantly increases for all lead times, especially for 1- to 6-month leads. For the *EPM* proposed here (*EPM*_*TD*+*EX*_), the hindcast skill further increases with 6- to 10-month leads because of the extratropical precursors, indicating that including the extratropical influence can significantly improve the ENSO forecast skill at a lead time of longer than 6 months. Moreover, the coefficients of *EP*_*WWV*_ (blue bars), *EP*_*OA*_ (red bars), and *EP*_*EX*_ (yellow bars) shown in Fig. 6c confirm the relative contributions of each predictor. For 1- to 3-month leads, the hindcast skill is mainly contributed by the ocean–atmosphere coupled feedback because the zonal surface wind change is almost simultaneous with the change of the Niño34 index, which is a typical feature of the Bjerknes feedback for the growth of ENSO events^6,7,9,53^. The contribution of the ocean–atmosphere coupled feedback decreases linearly with an increased lead-time. However, the contribution of eastward-propagating WWV reaches its maximum at a 3–5 months lead because the WWV propagation signal in the central Pacific leads the Niño34 index by 3–4 months^23^.

In contrast to the tropical precursors, the extratropical precursors have the largest contribution, with an 8- to 10-month lead. Because the lead-time extends over 6-months, the influence of the tropical dynamics is decreased with increased impacts of the extratropical precursors over both the North and South Pacific. We note that the coefficients of *EP*_*EX*_ are negative in the 1- and 2-month leads, which are possibly caused by the positive feedback between the PMM and ENSO. This result indicates that the PMM can trigger the central Pacific ENSO events^15^ while, on the interannual timescales^54^, it can also be enhanced from the tropical forcing. Therefore, extratropical atmosphere forcing may trigger ENSO events by increasing the zonal SST gradient across the western–central tropical Pacific and then modulate the anomalous westerlies along the equatorial Pacific. Thus, the extratropical teleconnection can effectively improve ENSO prediction with 8- to 10-month leads.

Figure 6d shows the correlation between the observed and predicted Niño34 index based on *EPM*_*TD*+*EX*_ and *EPM*_*TD*_ as a function of start month and lead time. Overall, when the hindcast lead time is less than 6-month, the prediction starting in boreal spring (March, April, and May: MAM) had the lowest correlation while that starting in boreal summer (JJA) tended to have the greatest hindcast skills, i.e., the typical SPB. We note that the prediction skill based on *EPM*_*TD*+*EX*_ (shading) showed a higher correlation than that based on *EPM*_*TD*_ (contours), specifically when the prediction started in MAM and the lead time was longer than 7-month. The SPB of ENSO events were mainly generated by a strong seasonal modulation of the growth rate^55^, indicating the difficulty in overcoming the SPB when only the tropical dynamics are considered as a precursor. The long-lead relationship between the extratropics and ENSO variability could partially reduce the long-lasting concern of SPB in the ENSO prediction.

**Figure 7:**
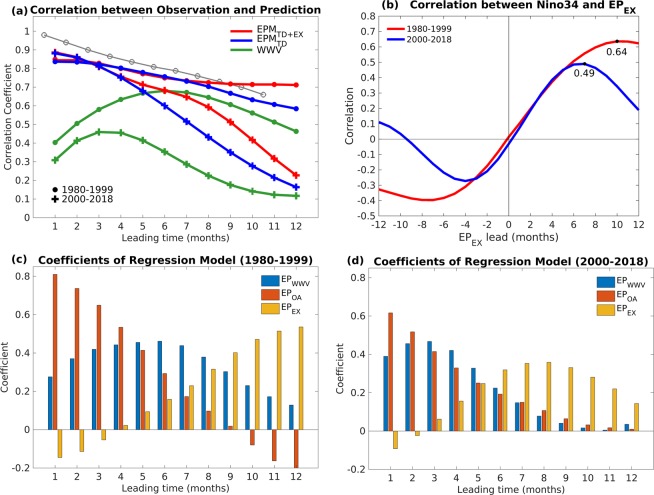
(**a**) Correlation between 3-month running mean observed and predicted Niño34 index based on the WWV index only (green curve), tropical dynamics only (*EPM*_*TD*_; blue curve), tropical and extratropical precursors together (*EPM*_*TD*+*EX*_; red curve) with different lead-times during 1980–1999 (dot-markers) and 2000–2018 (cross-markers). The prediction result of Clarke and Van Gorder (2003) based on WWV and Indo-Pacific winds for the period 1981–2001 is presented as grey curve with circle-markers. (**b**) Lead-lag correlation of Niño3.4 index with the EEMD filtered *EP*_*EX*_ during 1980–1999 (red curve) and 2000–2018 (blue curve). Maximum correlations are labeled (black dots). (**c**,**d**) Show the coefficients of the multivariate linear regression model contributed by *EP*_*WWV*_ (blue bars), *EP*_*OA*_ (red bars) and *EP*_*EX*_ (yellow bars) with different leading times during 1980–1999 and 2000–2018, respectively.

Previous studies found that the ENSO hindcast/forecast skill has been reduced for the first decade of the 21^st^ century^4,21^. Figure 7a shows the correlation between the observed and predicted Niño3.4 index based on three different schemes of the linear regression model with different lead times during 1980–1999 (dot-markers) and 2000–2018 (cross-markers). The difference in the ENSO prediction skill before and after 2000 can be easily observed. The maximum lead time for the WWV index (green curves) before 2000 (6 months) was longer than that after 2000 (3 months), which is consistent with previous studies that the WWV lead time changed from 6–9 months in 1979–1999 to 3–4 months after 2000^21,56^. Moreover, the hindcast skill (correlation) between the observed Niño3.4 and WWV index was much higher before 2000, which is consistent with the decadal change since 2000 that was shown in previous studies^21,23^. For the prediction model based on the tropical dynamics only (blue curves), the correlation between observation and prediction during 1980–1999 is also much higher. The correlation was 0.76 from 1980–1999 and only 0.60 from 2000–2018 with a 6-month lead, which is consistent with the decrease in the correlation between Niño3.4 and WWV indices after 2000^21,56^.

When the new prediction scheme (*EPM*) was considered based on both the tropical dynamics and extratropical teleconnection (Fig. 7a, red curves), the hindcast skill was improved both before and after 2000. Although the hindcast skill was higher before 2000, the improvement in the hindcast skill was greater in 2000–2018 compared with that during 1980–1999. The relationship between the Niño3.4 index and extratropical forcing also decreased after 2000, as shown in Fig. 7b, where the maximum lead time for the *EP*_*EX*_ index in 1980–1999 (10 months) is longer than that in 2000–2018 (7 months) and the corresponding correlation coefficient decreased from 0.64 to 0.49 after 2000. However, compared with WWV, the change in the extratropical forcing was relatively small and the prediction lead-time stayed at more than 7 months, and there was an improvement in the prediction skill after 2000.

To further investigate the change of relative contribution in *EPM*, the coefficient of *EP*_*WWV*_ (blue bars), *EP*_*OA*_ (red bars), and *EP*_*EX*_ (yellow bars) in different *EPM* lead-times before and after 2000 is shown in Fig. 7c,d. The maximum contribution of *EP*_*WWV*_ occurred with a 6-month lead in 1980–1999 and with a 3-month lead in 2000–2018. For 1980–1999, the contribution of the propagation of WWV to the hindcast skill was greater than the contribution of the extratropical atmospheric variability before the 8-month lead. On the other hand, the contribution of the extratropical forcing in 2000–2018 was larger than the contribution of the propagation of WWV after a 6-month lead, indicating the increased importance of the extratropics in predicting ENSO after 2000. The hindcast skill is mainly contributed by the ocean–atmosphere-coupled feedback for a short prediction lead time (1- to 2-month), and the importance of the ocean–atmosphere-coupled feedback decreased linearly with increased prediction lead-time. These results suggest that the extratropical atmospheric precursors in both the North and South Pacific are important in predicting ENSO and can reduce the long-lasting concern about SPB in the first 2 decades of the 21^st^ century.

To compare our *EPM* with other ENSO prediction models using tropical precursors, the prediction results from Clarke and Van Gorder^57^ based on the WWV and Indo-Pacific winds for the period 1981–2001 are shown in Fig. 7a. For a short lead time, the hindcast skill of their model was higher than that of *EPM* because they added eastern equatorial Indian Ocean wind stress to include the propagating signal of equatorial winds. However, when the lead time was longer than 8 months, the contribution of extratropical atmospheric precursors was more significant and the hindcast skill of *EPM* was higher than the model presented by Clarke and Van Gorder^57^.

Extratropical signals have been suggested to improve the ENSO prediction. Boschat’s regression model based on JFM tropical WWV and extratropical SST precursors achieved a higher correlation score than the model that uses the JFM tropical WWV and zonal wind stress only for 1979–2008 (0.72 compared to 0.61 correlation)^58^. Our correlation score of the *EPM* prediction from JFM based on tropical dynamics only (*EPM*_*TD*_) and tropical and extratropical precursors together (*EPM*_*TD*+*EX*_) was 0.66 and 0.74 for 1980–2008, respectively, which is comparable with Boschat’s results.

The hindcast correlation of statistical models in “IRI ENSO Forecast”, presented on the International Research Institute for Climate and Society (IRI) website (https://iri.columbia.edu/our-expertise/climate/enso/), is further shown in Fig. S2 for 2002–2018. The hindcast skill of most statistical models was lower than the *EPM* either based on the tropical dynamics and extratropical ocean–atmosphere (*EPM*_*TD+EX*_) or based on the tropical dynamics only (*EPM*_*TD*_). Overall, the proposed *EPM* combining the tropical dynamics and extratropical forcing significantly enhanced the ENSO prediction skill, particularly for a forecast with a long lead time. This suggests that the ENSO occurrences after 2000 may rely more on the ocean–atmospheric process resulting from the extratropical influence that was discussed earlier^1,15^.

The internal tropical dynamics, including the propagation of accumulated WWV via the recharge–discharge process and the ocean–atmosphere coupling, serve as a necessary precondition for the possible growth of ENSO events and they play a dominant role in the ENSO evolution^1,14,23^. Other remote precursors such as the extratropical atmosphere forcing are additional triggering forcing that could further suppress or enhance the final ENSO development through the equatorial ocean–atmosphere coupling^1,10,23^. When the sufficient preconditioned heat content exists at the equator, as determined by the tropical dynamics combined with the extratropical forcing, the tropical Pacific warming in Spring could develop into an ENSO event. However, the WWV was no longer a highly reliable precursor for ENSO after 2000 because of the La Niña-like background mean state and steeper equatorial thermocline tilt, which hampered the eastward propagation of WWV^1,59,60^. Thus, the role of WWV decreased and the ENSO prediction skill also declined. Some studies suggested that the variability of North Pacific atmosphere became more effective at initiating El Niño events after 1990s^15,61^. Moreover, the variance of the PMM associated with extratropical atmosphere forcing will increase in a warmer mean climate state^62^. These results supported the increasing importance of the extratropical precursors in predicting ENSO for the first 2 decades of the 21^st^ century and in a global warming situation.

## Data and Methods

### Observation and reanalysis data

In this study, several reanalysis datasets were used in constructing the statistical prediction model. Data from 1980 to the present were used. The ocean temperature data used in this analysis were the pentad global ocean data assimilation system (GODAS) on a 1/3° latitude × 1.0° longitude global grid^63^. The SST was defined as the temperature of the first layer of the ocean (taken at 5-m depth). The near-surface wind data (0.995-sigma level) and sea level pressure data from the National Centers for Environmental Prediction and the National Center for Atmospheric Research (NCEP–NCAR) reanalysis project^64^ on a 2.5° × 2.5° horizontal grid resolution were used to analyze the ocean–atmosphere coupling and extratropical atmospheric variability teleconnection. The anomalies here are based on the climatology from 1980 to 2018. *EP*_*WWV*_, *EP*_*OA*_, and *EP*_*EX*_ were estimated using pentad data (5-day average) and then averaged monthly to build the multivariate linear regression model. All comparisons are made on a monthly time scale.

### Definition of ENSO events

In this study, the El Niño (La Niña) was defined as an event with a 3-month running averaged Niño34 index that was greater than half a standard deviation (smaller than negative half a standard deviation). We note that some of the weak ENSO events defined here are slightly different from the definition of the Climate Prediction Center (CPC) (https://origin.cpc.ncep.noaa.gov/products/analysis_monitoring/ensostuff/ONI_v5.php) because different datasets and different periods of the climatological mean are used. We chose the observed ENSO events based on the consensus of the definitions here and by the CPC. During the analysis period, there were 12 El Niño events (1982/83, 1986/87, 1987/88, 1991/92, 1994/95, 1997/98, 2002/03, 2004/05, 2006/07, 2009/10, 2014/15, and 2015/16) and 14 La Niña events (1983/84, 1984/85, 1988/89, 1995/96, 1998/99, 1999/00, 2000/01, 2005/06, 2007/08, 2008/09, 2010/11, 2011/12, 2016/17, and 2017/18).

### ENSO prediction model

The ENSO prediction model (*EPM*) was built as follows:

2 $$\begin{array}{c}EPM(t+\varphi )=\frac{{\sigma }_{O}(m)}{{\sigma }_{P}(m)}({\beta }_{1}E{P}_{WWV}(t)+{\beta }_{2}E{P}_{OA}(t)+{\beta }_{3}E{P}_{EX}(t))\end{array}$$

The first two terms on the right-hand side are called “tropical dynamics”, including the predictors according to the propagation of WWV in the central Pacific (*EP*_*WWV*_) and the ocean–atmosphere feedback (*EP*_*OA*_); the third term, *EP*_*EX*_, is called the extratropical atmosphere forcing term. *EP*_*WWV*_, *EP*_*OA*_, and *EP*_*EX*_ were normalized before inputting into the linear regression model. The coefficients *β*_1_, *β*_2_, and *β*_3_ were derived from the multivariate linear regression analysis, and they indicate the linear association between the predictors and observations, which may change with a different lead time. The monthly scaling function, $\frac{{\sigma }_{O}(m)}{{\sigma }_{P}(m)}$, based on the ratio between the standard deviation of the observation (*σ*_*O*_) and the prediction (*σ*_*P*_) for the month (*m*) is applied to adjust the seasonal variance of prediction to observational variance (i.e., signal ratio). Then, a statistical prediction model is built to predict ENSO based on different lead times (*φ*) to evaluate the hindcast skill.

#### Extratropical ENSO precursors index

The extratropical ENSO precursor index is represented by a combination of SLPAs that were averaged over the significant positive and negative correlation centers, as shown in Fig. 2. Four significant regions were chosen because the others are dependent^28^; the red boxes from top are N1 (175°–120°W, 50°–75°N), N2 (180°–135°W, 10°–30°N), S1 (160°E–150°W, 25°–45°S), and S2 (180°–140°W, 50°–70°S). The index is defined as follows:

3 $$\begin{array}{c}E{P}_{EX}=(SLP{A}_{{\rm{N}}1}-SLP{A}_{N2})+(SLP{A}_{S2}-SLP{A}_{s1})\end{array}$$

An EEMD filter is performed to remove the high-frequency component while maintaining the interannual extratropical variability. In this study, the first ten IMFs are obtained for EEMD and only IMF_6–10_ of *EP*_*EX*_, which exhibits a lower frequency with a period from 12 months to 7 years, is used to represent the interannual component of *EP*_*EX*_.

#### Tropical ENSO precursors index

The tropical dynamics term *EPM* is constructed by a function of WWV in the central Pacific [*EP*_*WWV*_(*t*)] and the ocean–atmosphere feedback [*EP*_*OA*_(*t*)]^23^. The WWV function is defined based on the feature of eastward propagation WWV as follows:

4 $$\begin{array}{c}E{P}_{WWV}(t)={\alpha }_{1}D20{a}_{180^\circ }(t-5\,pentads)+{\alpha }_{2}D20{a}_{170^\circ W}(t-3\,pentads)+{\alpha }_{3}D20{a}_{155^\circ W}(t)\end{array}$$

D20a is the 20 °C thermocline depth (D20) anomaly that is averaged between 2°S and 2°N and *t* is a given prediction initial time in pentad. The subscripts represent the longitudinal locations along the equator. The choice of longitudes and timings in *EP*_*WWV*_ was based on the propagation of the eastward Kelvin wave at approximately 30°/month^23^. The weighting parameters *α*_1_ ∼ *a*_3_ are defined as the difference between the climatological mean of D20 at 175°E and the climatological mean of D20 at these three longitudes. *α*_1_, *α*_2_ and *α*_3_ are 2.8, 9.2 and 28.1 used here, respectively.

The other term, *EP*_*OA*_(*t*), measures the positive feedback resulting from the interaction between the atmosphere and ocean at the equator. *EP*_*OA*_(*t*) reflects that the ocean–atmosphere coupled feedback can amplify or suppress the anomaly of *EP*_*WWV*_(*t*), according to the events of westerly wind events/easterly wind surges (WWEs/EWSs). This coupled feedback is constructed as:

5 $$\begin{array}{c}E{P}_{OA}(t)=sign(event(t,{w}_{x}))\cdot dH(t,E{P}_{WWV})\end{array}$$

where *w*_*x*_ represents the zonal surface wind anomalies over the region 180°–155°W, 2°S–2°N. The *EP*_*OA*_(*t*) includes two parts. The first part is to define the wind event (westerly or easterly anomalies) as follows:

6 $$\begin{array}{c}event(t,{w}_{x})=\left\{\begin{array}{c}positive\,{w}_{x}\,in\,[t-10\,pentads,t]\\ negative\,{w}_{x}\,in\,[t-10\,pentads,t]\end{array}\right.\end{array}$$

The second part is to measure the modulation of the wind event on the change of WWV, which can amplify or suppress the development of the Bjerknes feedback. Here, we assume that *EP*_*OA*_(*t*) is active only when the *EP*_*WWV*_ changes dramatically and the same sign wind events can be detected for more than 5 pentads (25 days) during a continuous period of 10 pentads (50 days), representing the consistent westerly or easterly anomalies. The modulation of the wind event is defined as:

7 $$dH(t,E{P}_{WWV})=\left\{\begin{array}{cc}\frac{|E{P}_{WWV}(t)-E{P}_{WWV}(t-5)|+|E{P}_{WWV}(t-5)-E{P}_{WWV}(t-10)|}{2}, & 5\,pentads\le event(t,{w}_{x})\\ 0, & event\,(t,{w}_{x}) < 5\,pentads\end{array}\right.$$

### Ensemble empirical mode decomposition (EEMD)

Empirical mode decomposition (EMD) is a method of breaking down signals into various components^65^. The decomposition is designed to seek the different intrinsic mode functions (IMFs) of oscillations from high-frequency to low-frequency based on local time scales. After extracting *M*-1 IMFs via EMD, the original signal can be reconstructed by superposing the obtained IMFs and the residual (*r*_*M*_):

8 $$\begin{array}{c}X(t)=\mathop{\sum }\limits_{m=1}^{M-1}{{\rm{IMF}}}_{m}(t)+{r}_{M}(t)\end{array}$$

A higher order of IMF (larger *m*) refers to a lower frequency component.

Ensemble EMD (EEMD) is an extension of the EMD^66,67^. The IMFs from EEMD are based on an average of EMD ensembles, and each EMD member has an added independent white noise with the same standard deviation. In contrast to many almost previous decomposition methods, the EEMD is empirical, intuitive, direct, and adaptive. Specifically, no pre-determined basis functions are required.

Using the EEMD as a low-pass filter is natural and it is more practical in real-time forecast than the conventional time filters. The potential end-point effect issue can be minimized. The end-point effect occurs at the beginning and end of the signal because there is no point before the first data point and after the last one to be considered^68^. A modified linear extrapolation method^66^, where additional extrema are added to the ends of the data by linear extrapolation of the two maxima (minima) near the end point, is adopted to reduces the end-point effect.

## Supplementary information

Supplementary information.

## Data Availability

The data supporting the findings of this study are available within the article. Any other data are available from the corresponding author upon request.
