# Supplementary material for: Enhancing the ENSO Predictability beyond the Spring Barrier
Source: Sci Rep. 2020 Jan 22;10:984. doi: 10.1038/s41598-020-57853-7 (PMC6976663; doi:10.1038/s41598-020-57853-7)
Supplement: Supplementary file 1 — Supplementary information. [file 41598_2020_57853_MOESM1_ESM.docx]

| 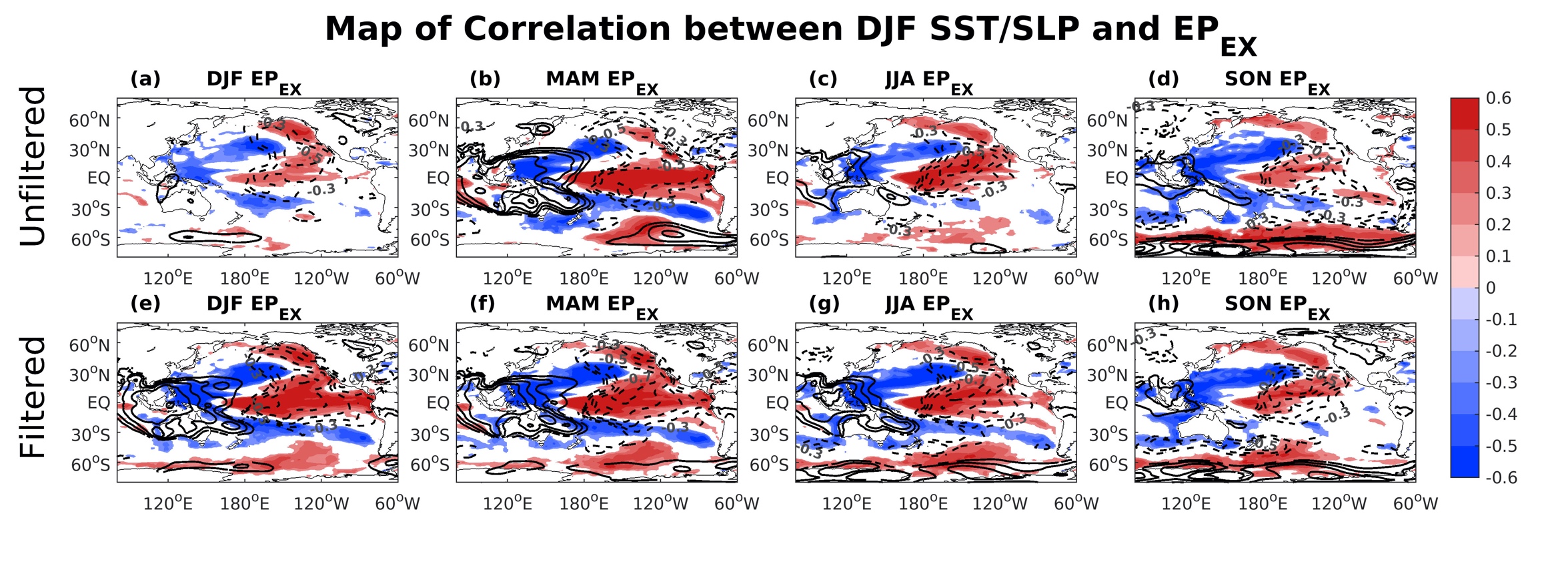 |
| --- |
| Figure S1. Correlation between the (a-d) unfiltered and (e-h) EEMD filtered EP_EX_ during the DJF, MAM, JJA, and SON and the following DJF SST (shading) and SLP (contours) anomalies. |

| 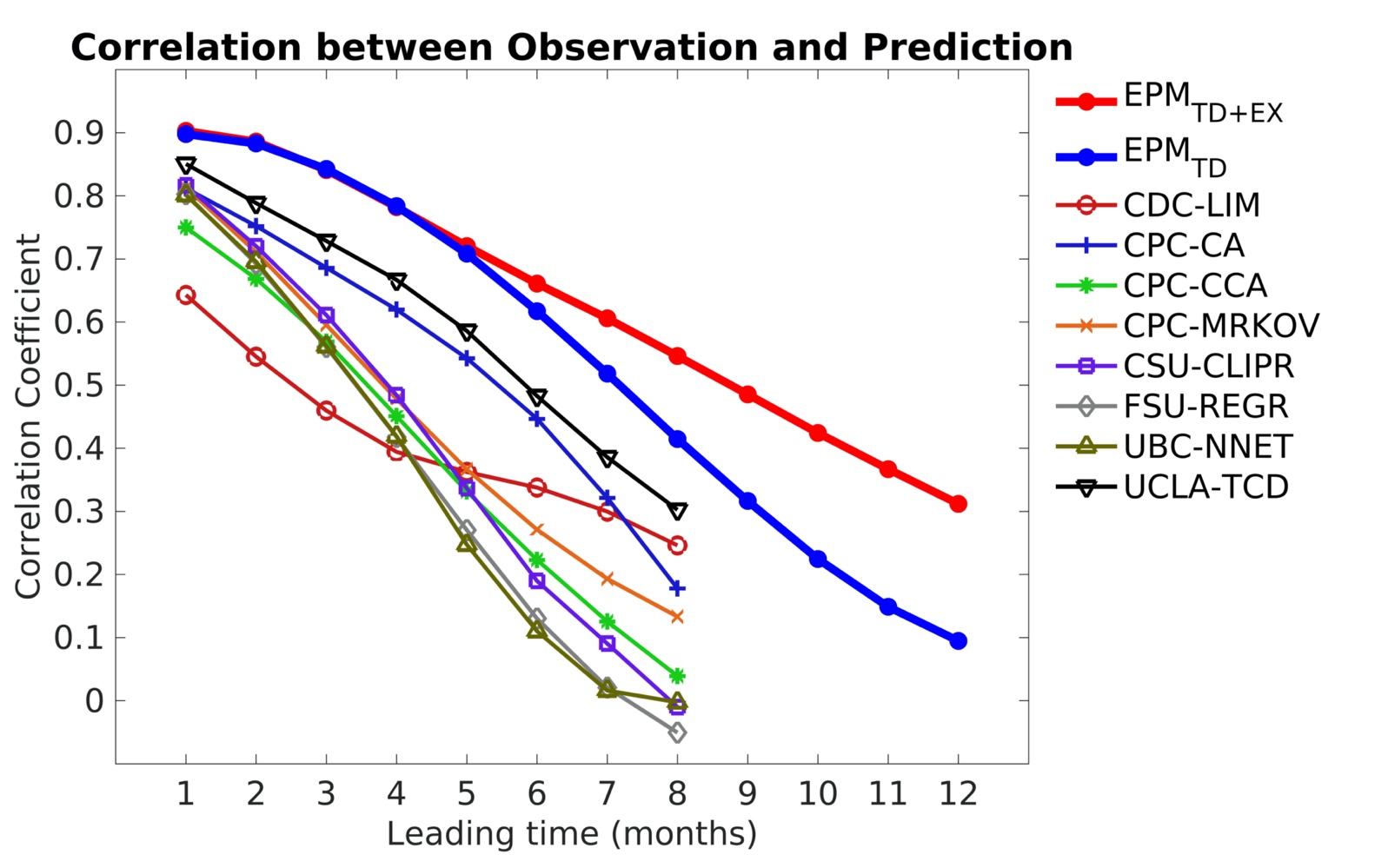 |
| --- |
| Figure S2. Correlation between 3-month running mean observed and predicted Niño34 index based on the tropical dynamics only (${EPM}_{TD}$; blue curve), tropical and extratropical precursors together (${EPM}_{TD+EX}$; red curve) during 2002-2018. The correlation between observations and statistical model forecasts presented in IRI ENSO plume are shown with thin curves and different markers. |
